# Analysis of Goldman Applanation Tonometry with and without fluorescein among glaucomatous and nonglaucomatous patients attending Mzuzu Central Hospital in Malawi: A cross‐sectional study

**DOI:** 10.1002/hsr2.1390

**Published:** 2023-07-01

**Authors:** Tryness Munyapa, Thokozani Mzumara, Grace Ogbonna, Augustine Mvula

**Affiliations:** ^1^ Department of Optometry Mzuzu University Mzuzu Malawi; ^2^ Department of Ophthalmology Mzimba North District Hospital Mzuzu Malawi

**Keywords:** cornea, fluorescein, glaucoma, goldmann applanation tonometry, intraocular pressure

## Abstract

**Background and Aim:**

Goldman Applanation Tonometry (GAT), the gold standard of tonometry, is used without fluorescein in low‐resource settings. Nevertheless, corneal biomechanics differ among population groups.

**Purpose:**

The aim of the study is to assess the relationship between GAT findings with and without fluorescein among glaucomatous and nonglaucomatous adults in Malawi.

**Methods:**

This was a cross‐sectional quantitative study involving 22 glaucoma patients and 22 nonglaucoma patients at Mzuzu Central Hospital. We used a purposive sampling technique to select participants into the two groups. Next, we measured intraocular pressure using GAT with and without fluorescein. Then we entered the data into SPSS version 25. We employed the Wilcoxon test to make comparisons based on age and gender. We considered the value of *p* < 0.05 statistically significant.

**Results:**

There is a statistically significant and strong positive correlation between nfGAT and fGAT among both glaucoma (*r* = 0.989, *p* < 0.001) and nonglaucoma (*r* = 0.955, *p* < 0.001). According to age, there is no significant difference in IOP value measured with nfGAT and fGAT for both glaucomas (*p* = 0.109) and nonglaucoma subjects (*p* = 0.076). However, significant differences were observed between nfGAT and fGAT mean IOP according to sex among both glaucomatous and nonglaucomatous subjects (*p* = 0.017 and *p* = 0.32, respectively).

**Conclusion:**

The study suggests that the merits of intraocular pressure measured using GAT without fluorescein are not speculative, therefore the two techniques can be routinely used interchangeably in diagnosing and managing glaucoma.

## 1 INTRODUCTION

Normal intraocular pressure (IOP) is critical for the maintenance of pathophysiological and physiological processes of the eye mainly by providing structural support. The equilibrium between aqueous secretion and drainage results in normal IOP, which ranges from 10 to 21 mmHg.^1^ Elevation in IOP above the normal range leads to ocular hypertension, which in many instances, may cause glaucoma.^2^ The prevalence of glaucoma ranges from 2.9% in Europe to 4.7% in Africa.^3^

Glaucoma is a group of disorders related to optic neuropathy characterized by optic nerve damage due to the loss of ganglion cells.^4^ The development of glaucoma and the progression of glaucomatous damage is strongly linked to visual loss.^5^ Glaucoma is the leading cause of irreversible blindness in sub‐Saharan Africa.^6^ Nevertheless, 50% of glaucoma cases remain undiagnosed.^7^ The key to the management of glaucoma lies in strategies aimed at enhancing screening for early detection and management of glaucoma.^8^ To diagnose and monitor glaucoma, practitioners utilize methods such as optical coherence, perimetry, and IOP measurement.^9^ Reliable measurement of intraocular pressure, otherwise known as tonometry, is crucial to the management of glaucoma since IOP is the only modifiable risk factor for glaucoma progression with time.^10^

Goldman Applanation Tonometry (GAT) remains the gold standard for measuring IOP.^11^ Based on the Imbert–Fick law, GAT measures the force required to flatten a 3.06 mm area of the central cornea.^4^ Usually, the procedure involves the addition of fluorescein dye to enhance the visualization of the mires.^11^ Although fluorescein as a dye enhances the visualization of mires, it is not without its challenges in clinical practice. First, the use of the dye has not been standardized such that too much or too little fluorescein affects the diameter of the mires leading to under‐ or overestimation of IOP, respectively.^4^ Moreover, in rural settings of most low‐income countries, fluorescein may not be readily available. This has prompted some researchers to try GAT without fluorescein (nfGAT).^12^

A previous study reported that the merits of using GAT without fluorescein are speculative.^13^ However, reports from Sudan^10^ found statistically significant differences in GAT with and without fluorescein. Again, researchers^11^ in Germany found that GAT values without fluorescein were lower compared with GAT with fluorescein dye. However, Bamdad and colleagues assessed the difference in GAT measurements with and without fluorescein among nonglaucoma patients in Iran and found no statistically significant difference.^12^ Again, others^4^ in Turkey found no significant difference between nfGAT with fGAT.

Considering that GAT is dependent on corneal properties,^14^, ^15^ it is imperative to suggest that IOP measurements may be influenced by demographic factors such as race, age, and gender.^16^ In general, Africans have thinner corneas however differences also exist among ethnic groups within the African continent.^17^ In part, the differences can be explained by ethnic variations.^18^ To the best of our knowledge, there is no study that has examined the relationship between the two techniques among the Malawian population.

In Malawi, Glaucoma is the second leading cause of blindness, however, glaucoma poses a greater public health challenge since it is more difficult to diagnose.^18^ Therefore, this paper aims at analyzing the relationship between GAT with fluorescein and without fluorescein among glaucoma and nonglaucomatous Malawians. This information is useful for eye care practitioners in low‐resourced countries in the fight against irreversible blindness caused by glaucoma.

## 2 METHODS

This was a cross‐sectional study conducted among patients attending Mzuzu Central Hospital's Ophthalmology Outpatient department. Participants were selected using a purposive sampling technique. The participants comprised 22 glaucomatous and 22 nonglaucomatous participants. Participants aged 18 years and older were included in this study. Furthermore, participants with corneal opacities, those taking systemic steroids, and pregnant women were excluded.

### 2.1 Procedures

After obtaining informed consent, we conducted an ophthalmologic screening which includes case history taking, visual acuity testing, and slit lamp examination to determine whether participants met the eligibility criteria. IOP was measured using a Slit lamp‐mounted Godmann applanation tonometer. The nfGAT was measured first before fGAT.

To measure IOP with nfGAT, white light was focused on the temporal probe and perpendicular to the tonometer. The examiner observed the inner edges of the semicircle mires and adjusted them until they are in contact.^4^ Three nfGAT measurements were taken. After an interval of 5 min, Fluorescein Sodium Ophthalmic Strips were applied inside the participant's eyes, and the same procedure was done under cobalt blue light. Similarly, three measurements were taken for the fGAT. Finally, the cornea was examined using fluorescein and cobalt blue light to rule out corneal abrasions caused by the examination. To avoid the effect of diurnal variations, all IOP measurements were done between 9:00 and 11:00 a.m. All measurements were conducted by a single examiner to avoid inter‐examiner variations which may have influenced results.

### 2.2 Analysis

Data were entered into SPSS version 25. Descriptive statistics were employed to determine the distribution of the measurements while inferential statistics were used to test for correlation among variables. Data were grouped based on age (18–39, 40–59, 60–79, and ≥80 years) and gender (male and female). Furthermore, we computed the difference between the two measurements. Comparisons of nfGAT and fGAT IOP among glaucomatous and nonglaucomatous participants were done using the independent and Wilcoxon paired *t* test. Pearson correlation coefficient test was conducted to evaluate the relationship between nfGAT and fGAT measurements. In addition, we employed the intraclass correlation coefficient to assess agreement between the two methods. A *p* < 0.05 was considered statistically significant. All statistical tests were two sided tests.

## 3 RESULTS

### 3.1 Demographic characteristics of study participants

The results of the study showed that 47.7% of the participants were males (*n* = 21/44) while 52.3% were females (*n* = 23/44). The mean age of the participants was 51.1 ± 22.5 (range; 18–91 years). The mean age for males was 58.4 ± 21.3 and for females was 44.4 ± 22.0. An independent *t* test depicted that the mean difference in age between sex was statistically significant *t* (86) = 3.0327, *p* = 0.0032. According to age, the majority of the participants belonged to the 18–39 years' age group (*n* = 16), 36.1% and the minority age group was ≥80 years (Table 1).

**Table 1:** Demographic characteristics of the study participants.

Age groups	Males	Females
All participants	21 (47.7%)	23 (52.3%)
18–39 years	6 (13.4%)	10 (22.7%)
40–59 years	3 (6.8%)	7 (15.9%)
60–79 years	9 (20.5%)	5 (11.4%)
≥80 years	3 (6.8%)	1 (2.2%)

	Mean (SD)	Range
Age	51.1 ± 22.5	18–91

	Mean IOP (nfGAT)	Mean IOP (fGAT)
Glaucoma	17.55 ± 5.64	18.25 ± 5.80
Nonglaucoma	13.34 ± 1.48	13.60 ± 1.22

According to sex, the glaucoma group had 12/22 (54.5%) males and 10/22 (45.5%) females while the nonglaucoma group had 9/22 (40.9%) males and 13/22 (59.1%) females, however, a *χ* ^2^ test showed that there is no statistically significant association between sex and glaucoma (*p* = 0.365). Regarding age group, the majority 10/22 (45.5%) of glaucoma participants were between the age group 60–79 years and the least frequent age group was 18–39 years 2/22 (9.1%) while in the nonglaucoma group, the most common age group was 18–39 years 14/22 (63.6%) and the least frequent was 80 years and above 1/22 (4.5%). Pearson's *χ* ^2^ test showed that there is a statistically significant association between having glaucoma and age (*p* = 0.003).

### 3.2 Distribution of GAT values

In general, the average mean IOP without fluorescein was lower 15.44 (SD = 4.598) mmHg compared with GAT with fluorescein at 15.92 (SD = 4.76) mmHg. A paired *t* test indicated that the difference was statistically significant (*p* < 0.001). According to the study group, the mean IOP reading among glaucomatous subjects was 17.55 ± 5.64 mmHG with nonfluorescein GAT (nfGAT) and 18.25 ± 5.80 mmHg with fluorescein GAT (fGAT). Paired *t* test showed that the difference was statistically significant (*p* = 0.001). On the other hand, the mean IOP among nonglaucomatous subjects was 13.34 ± 1.48 mmHg with nfGAT and 13.60 ± 1.22 mmHg with fGAT. The paired *t* test showed a statistically significant difference between the two means (*p* = 0.020).

### 3.3 Agreement between fGAT and nGAT

A one sample *t* test was conducted on the difference between the two measures as a prerequisite for Bland–Altman test. The mean was −0.47 (SD = 0.73) confidence interval (CI) (0.7, 0.2) and it was statistically significant therefore they cannot show a useful level of agreement (*t*(43)= −4.317, *p* < 0.001). Nevertheless, the intraclass correlation coefficient applying a two way mixed model and absolute agreement showed an excellent reliability between the two methods. The average measure was 0.994 with a 95% CI from 0.989 to 0.997.

### 3.4 Relationship between fGAT and nGAT

**Figure 1:**
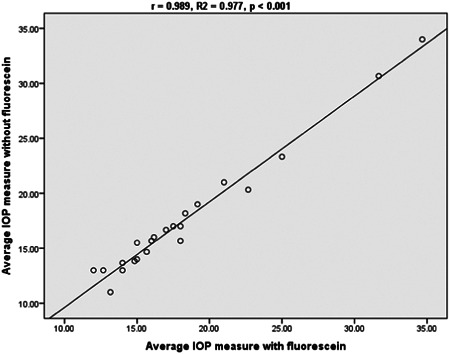
Correlation of nfGAT and fGAT among glaucomatous subjects. Relationship between IOP with and without fluorescein among glaucomatous subjects. fGAT, fluorescein GAT; IOP, normal intraocular pressure; nfGAT, nonfluorescein GAT.

**Figure 2:**
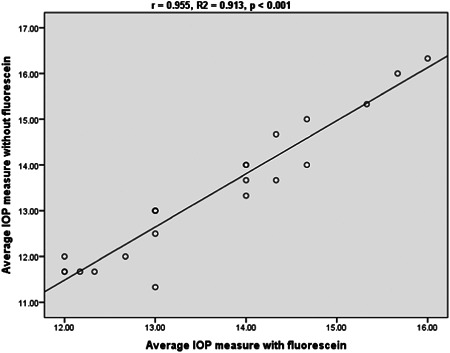
Correlation of nfGAT and fGAT among nonglaucomatous subjects. Relationship between IOP with and without fluorescein among nonglaucomatous subjects. fGAT, fluorescein GAT; IOP, normal intraocular pressure; nfGAT, nonfluorescein GAT.

A Pearson correlation coefficient test depicted a statistically significant strong positive correlation in IOP measurement between the Glaucomatous group (*R* = 0.989, *R*2 = 0.977, *p* < 0.001) and the nonglaucomatous group (*R* = 0.955, *R*2 = 0.913, *p* < 0.001) (Figures 1 and 2).

### 3.5 Relationship between nfGAT and fGAT according to sex

**Figure 3:**
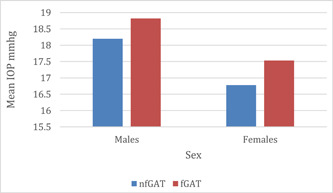
Mean IOP against sex for nfGAT and fGAT in glaucomatous subjects. Relationship between nfGAT and fGAT according to sex. fGAT, fluorescein GAT; IOP, normal intraocular pressure; nfGAT, nonfluorescein GAT.

Among male glaucomatous subjects, mean IOP was 18.20 ± 7.17 mmHg with nfGAT and 18.82 ± 7.16 mmHg with fGAT. Wilcoxon test showed statistically significant difference between the two mean IOPs (*p* = 0.017), with mean fGAT IOP being high. In female glaucomatous subjects, mean IOP of 16.78 ± 3.18 mmHg for nfGAT and 17.57 ± 3.87 mmHg for fGAT. Results showed a significant difference between nfGAT and fGAT IOP (*p* = 0.032), with fGAT mean IOP being higher than nfGAT (Figure 3).

Among nonglaucomatous male subjects, mean IOP was 13.63 ± 1.48 mmHg with nfGAT and 13.81 ± 1.23 mmHg with fGAT. Wilcoxon test showed that there was no significant difference between the two IOP measurements (*p* = 0.172). However, among nonglaucomatous female subjects there was significant difference between nfGAT mean IOP which was 13.14 ± 1.48 mmHg, and fGAT mean IOP which was 13.45 ± 1.23 mmHg (*p* = 0.049). The Wilcoxon test results among glaucomatous subjects showed statistically significance difference between nfGAT and fGAT (*p* = 0.049) (Table 2).

**Table 2:** Wilcoxon matched‐paired test for mean IOP between nfGAT and fGAT in nonglaucomatous subjects based on sex.

Sex	Mean IOP nfGAT	Mean IOP fGAT	*p* Value
Males	13.63 ± 1.48	13.81 ± 1.23	0.172
Female	13.14 ± 1.48	13.45 ± 1.23	**0.049** *

Abbreviations: fGAT, fluorescein GAT; IOP, normal intraocular pressure; nfGAT, nonfluorescein GAT.

* *p*‐value is statistically significant.

### 3.6 Relationship between nfGAT and fGAT according to age

**Figure 4:**
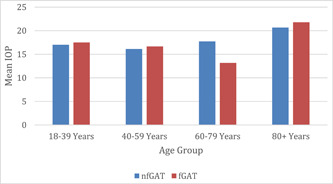
Mean IOP according to age for nfGAT and fGAT in glaucomatous subjects. Relationship between nfGAT and fGAT according to age among glaucomatous. fGAT, fluorescein GAT; IOP, normal intraocular pressure; nfGAT, nonfluorescein GAT.

**Figure 5:**
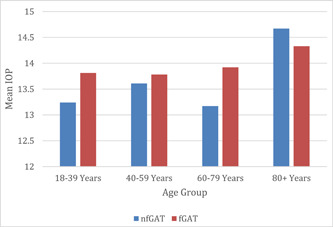
Mean IOP according to age group for nfGAT and fGAT in nonglaucomatous subjects. Relationship between nfGAT and fGAT according to age among nonglaucomatous. fGAT, fluorescein GAT; IOP, normal intraocular pressure; nfGAT, nonfluorescein GAT.

Among glaucomatous subjects, Wilcoxon matched‐pair tests significant difference between the two tests were only observed in age group 60–79 years (*p* = 0.04) (Figure 4). Among nonglaucomatous subjects, none of the age groups showed a significant difference between nfGAT and fGAT IOP (all *p* > 0.05) (Figure 5).

## 4 DISCUSSION

Glaucoma is the leading cause of irreversible blindness worldwide accounting for 6.6% of the world's blindness.^19^, ^20^ Several methods exist for measuring IOP, however, GAT is the gold standard.^21^ The results of the study showed that there is a strong correlation between IOP measured using fGAT and nGAT among the different study groups. This study provides a first overview of the distribution of IOP among the Malawian population and provides a reference for the population group.

Different IOP values have been reported among different populations and geographical locations.^3^ The mean IOP in this study is similar to a previous study in Sweden.^16^ However, it was lower than previously reported in Iran.^1^, ^22^ This implies that there is variability in the mean IOPs in the various studies possibly due to differences in the biometric distribution that can be seen in people from different ethnic groups.^14^, ^15^

In this study, IOP was positively correlated with age contrary to previous reports.^1^ Our study results can be explained by the unconventional outflow of aqueous through the ciliary body which accounts for 50% of aqueous outflow in healthy young adults and declines with age.^23^ Our findings highlight age as the risk factor for developing glaucoma and therefore emphasize routine screening for adults.

The majority of the glaucoma subjects in this study were male similar to the previous study at Queen Elizabeth Central Hospital in southern Malawi.^18^ Male predominance in health‐seeking is not uncommon in Africa where women lag behind in terms of accessing healthcare as a factor to various sociodemographic and cultural factors. Integration of glaucoma screening programs within mainstream gender‐sensitive services such as mothe–child health would help reach a lot of women, especially those living in rural communities.

The current study also found that there is no significant difference in GAT values measured with and without fluorescein similar to previous studies.^4^, ^12^ On the contrary, others^10^ found a statistically significant difference in GAT measured with and without fluorescein among both glaucoma and nonglaucomatous patients in Sudan. In addition, Arend and colleagues^11^ found that there was a difference between GAT with fluorescein and without. We attribute the variation to different races in the studies. However, the results of the current study imply that GAT without fluorescein can be used for both screening and diagnostic purposes among people within the study's geographical location.

The current study found no significant difference in GAT values measured with fGAT and nfGAT among different age groups except for 60–79 years old glaucomatous group. These findings disagree with what was reported by other authors^12^ who also found no significant difference between nfGAT and fGAT based on age in Iran. Noteworthy, the study in Iran used a narrower age range compared with the current study which recruited a wider age range (19–91 years). Indeed, the current study findings can be attributed to the fact that majority of glaucoma subjects were aged between 60 and 79 years. This suggests that age has a significant influence on differences in IOP measurements taken with nfGAT and fGAT.

According to sex, the current study has found significant differences between IOP measurements taken using nfGAT and fGAT in both glaucomatous and nonglaucomatous subjects. IOP measured with nfGAT was higher in both males and females, which implies that nfGAT and fGAT cannot be used interchangeably. However, the difference is likely to be insignificant clinically, as noted in a previous study.^24^ The findings are contrasting many studies. For example, a recent study^4^ reported no significant difference in IOP measurements between nfGAT and fGAT in both males and females. Similarly, Elzein and Saleem,^10^ in their hospital‐based study noted that IOP measured with and without fluorescein differed significantly with other variables but not with sex. Further contrary findings were reported by Bamdad and Vatan,^12^ who measured IOP in healthy subjects and reported no sex differences in IOP measurements between with‐fluorescein and without‐fluorescein GAT. The findings established by the current may partly be attributed to differences in the age distribution among males and females, as the majority of males were aged older while females were largely younger.

### 4.1 Limitations

The paper is not without drawbacks. First, our study employed a smaller sample size which might not be a full representation of the Malawian population. In addition, our study was hospital‐based and hence could be confounded by health‐seeking behavioral factors inherent to the socioeconomic status of the country of study. Moreover, our study did not assess systemic predictors such as heart rate, Body mass index, and blood pressure which could have confounding effects on the parameter. Nevertheless, the strongest caveat of our study is the wider age range which accounts for the effect of age on the development of glaucoma. Furthermore, our study establishes the distribution of IOP values for practitioners to use.

## 5 CONCLUSION AND RECOMMENDATIONS

The study concludes that there is a direct correlation between nGat and fGAT among the study population irrespective of their glaucomatous status, age, and gender. This therefore suggest that in the event of fluorescein shortages nGAT is a good alternative to fGAT using the white light on a slit among the study population. Nonetheless, we recommend that further studies with a larger sample size and community‐based study design to fully elucidate this phenomenon among this populace.

## AUTHOR CONTRIBUTIONS

**Tryness Munyapa**: Conceptualization; formal analysis; investigation; methodology; visualization; writing—original draft; writing—review and editing. **Thokozani Mzumara**: Data curation; formal analysis; investigation; methodology; supervision; visualization; writing—original draft; writing—review and editing. **Grace Ogbonna**: Conceptualization; formal analysis; methodology; supervision; visualization; writing—original draft; writing—review and editing. **Augustine Mvula**: Formal analysis; methodology; visualization; writing—original draft; writing—review and editing.

## CONFLICT OF INTEREST STATEMENT

The authors declare no conflict of interest.

## ETHICS STATEMENT

Our study was approved by the Mzuzu University Faculty of Health Sciences research committee (Ethical Clearance Nuumber FOHS/REC/21/087). The study followed the Helsinki Declaration such that informed consent was obtained from all participants. No participant was harmed during the study.

## TRANSPARENCY STATEMENT

The lead author Grace Ogbonna affirms that this manuscript is an honest, accurate, and transparent account of the study being reported; that no important aspects of the study have been omitted; and that any discrepancies from the study as planned (and, if relevant, registered) have been explained.

## Data Availability

The data is available upon request from the corresponding author. All authors have read and approved the final version of the manuscript [Grace Ogbonna] had full access to all of the data in this study and takes complete responsibility for the integrity of the data and the accuracy of the data analysis.
